# Morphology of the limbs in the semi-fossorial desert rodent species of *Tympanoctomys* (Octodontidae, Rodentia)

**DOI:** 10.3897/zookeys.710.14033

**Published:** 2017-10-19

**Authors:** M. Julieta Perez, Ruben M. Barquez, M. Monica Diaz

**Affiliations:** 1 PIDBA (Programa de Investigaciones de Biodiversidad Argentina), PCMA (Programa de Conservacion de los Murcielagos de Argentina), CONICET (Consejo Nacional de Investigaciones Cientificas y Tecnicas), Facultad de Ciencias Naturales e IML-Universidad Nacional de Tucuman. Miguel Lillo 251, 4000. Tucuman, Argentina; 2 Fundacion Miguel Lillo. Miguel Lillo 205, 4000. Tucuman, Argentina

**Keywords:** Argentina, Chalchalero Vizcacha Rat, description, morphology

## Abstract

Here, a detailed description of the forelimbs and hindlimbs of all living species of the genus *Tympanoctomys* are presented. These rodents, highly adapted to desert environments, are semi-fossorial with capacity to move on the surface as well as to build burrows. The shape, structure, and size of the limbs are described. Contrary to what was expected for scratch digging semi-fossorial species, *Tympanoctomys* have slender humerus, radius and ulna; with narrow epicondyles of the humerus and short olecranon of the ulna with poorly developed processes. Following our descriptions, no intrageneric morphological variation regarding to the configuration of the limbs was detected, probably due to phylogenetic proximity, and not related to specific variations in response to different use of substrates or habits. The obtained results constitute a source of previously unpublished information as well as an important base for future analysis in different studies, such as morphometric, morpho-functional, or phylogenetic researches.

## Introduction

*Tympanoctomys* is member of a clade of the family Octodontidae, restricted to Argentina (Reig 1989; Ojeda et al. 2013; Diaz et al. 2015). Octodontid rodents are highly diverse in eco-morphological aspects, including six genera with terrestrial, semi-fossorial, fossorial and subterranean forms (Ojeda et al. 2013; Verzi et al. 2015). Due to their broad ecological and geographical diversity at both sides of the Andean mountain range, these rodents represent a very interesting group in biogeographical and evolutionary aspects (Lessa et al. 2008; Ojeda 2010; Ojeda et al. 2013).

It was estimated that *Tympanoctomys* diverged about six million years ago (Gallardo and Kirsch 2001) in coincidence with the origin of deserts at Late Miocene (Gallardo et al. 2006); but other authors mentioned different times of divergence from 2.5 to 6.5 million years (see Upham and Patterson 2012; Gallardo et al. 2013; Upham and Patterson 2015; Verzi et al. 2016; Suarez-Villota et al. 2016; Alvarez et al. 2017). This genus is one of the few mammals (all rodents) most highly adapted to desert environments (Mares 1975, 1993; Bozinovic and Contreras 1990; Ojeda et al. 1996; Mares et al. 2000; Honeycutt et al. 2003). *Tympanoctomys* is a polytypic genus containing four living species: *T. aureus*, *T. barrerae*, *T. kirchnerorum*, and *T. loschalchalerosorum*, of which two species were originally described as separate genera (*Pipanacoctomys aureus* and *Salinoctomys loschalchalerosorum*) and *T. kirchnerorum* was recently described from the central Patagonia of Argentina (see Diaz et al. 2000; Barquez et al. 2002; Diaz and Verzi 2006; Gallardo et al. 2004, 2013, 2009; Teta et al. 2014; Diaz et al. 2015). Recently Suarez-Villota et al. (2016) tested the monophyly of these species through a molecular phylogenetic, and the analysis supported the notion that *Salinoctomys* and *Pipanacoctomys* are not distinct from *Tympanoctomys*. These results are in accordance with previous morphological and molecular researches where *Pipanacoctomys* is placed external to *Tympanoctomys*-*Salinoctomys* (Barquez et al. 2002; Upham and Patterson 2015, 2016).

*Tympanoctomys* is a small- sized octodontid, with body mass ranging from 67–104 g, head and body length from 130–170 mm (Mares et al. 2000; Teta et al. 2014); *T. aureus* is the largest species in the genus, with head and body length mean of 170 mm, *T. barrerae* is a middle size, with head and body length mean of 145 mm, only slightly larger than *T. loschalchalerosorum* (144–156 mm), and finally, *T. kirchnerorum* is the smallest, with head and body length mean of 129.4 mm (Verzi et al. 2015). They are endemic to arid regions of central and western Argentina, within the Monte, Chaco, and Patagonian Desert biomes, inhabiting salt basins, sand dunes, and open scrubland in Catamarca, Chubut, La Rioja, Mendoza, San Juan, La Pampa, and Neuquen provinces (see Verzi et al. 2015). Ecological aspects of the species are poorly known, *T. barrerae* being the best studied. The colonization success and expanded range of *T. barrerae* has been suggested to be the result of a set of behavioral, ecomorphological, and physiological features that allows better utilization of salt basin, open xeric habitats, and hypersaline food resources (Mares et al. 1997; Ojeda et al. 1999; Gallardo et al. 2007; Ojeda et al. 2013).

Members of the genus *Tympanoctomys* have the capability to move on the surface as well as to build complex burrows (Ojeda et al. 2013; Teta et al. 2014; Diaz et al. 2015), a feature that distinguishes the semi-fossorial forms (Eisenberg 1981). According to Lessa et al. (2008), this digging ability is not reflected in substantial changes in the locomotor system of subterranean octodontids, strongly suggesting that during the early evolution of these rodents, behavioral events have preceded and probably promoted subsequent morphological changes. In addition, among fossorial rodents two burrow strategies have been developed: with the incisors (chisel-tooth digging) or with the forelimbs (scratch-digging), while they can use both types or only one of them. The cranio-dental morphology of *Tympanoctomys* is not similar to that of those corresponding to chisel-tooth digging as the case of *Spalacopus*, subterranean octodontid (Vassallo and Verzi 2001; Bacigalupe et al. 2002; Verzi 2002; Olivares et al. 2004; Lessa et al. 2008; Becerra et al. 2012). Based on this idea, some authors have analyzed skeletal features in few related genera and members of the family, but including only one species of *Tympanoctomys* (*T. barrerae*), proposing novel functional inferences (Morgan and Verzi 2006; Lessa et al. 2008; Morgan 2009; Morgan and Verzi 2011; Morgan and Alvarez 2013).

However, studies of postcranial anatomy of this genus are insufficient. The objective of our research was to describe in detail the morphology of the forelimbs and hindlimbs of all species of the genus *Tympanoctomys*, mainly analyzing shape and size of some elements. Expecting to generate new data set that allow infer correlation between postcranial morphology and digging strategy.

## Materials and methods

Sixty specimens of all the living species of the genus *Tympanoctomys* were examined: *Tympanoctomys aureus* (22), *Tympanoctomys barrerae* (35), *Tympanoctomys kirchnerorum* (2), and *Tympanoctomys loschalchalerosorum* (1). All with complete postcranial skeletons deposited in three argentine collections, CML (Coleccion Mamiferos Lillo; Universidad Nacional de Tucuman, Tucuman), CNP (Coleccion de Mamiferos “Elio Massoia”, Centro Nacional Patagonico, Puerto Madryn, Chubut), and CMI (Coleccion de Mamiferos IADIZA, Mendoza). Also, four individuals of *Ctenomys opimus* were measured for comparisons with the species of *Tympanoctomys*. The specific localities and collection numbers of specimens are given in Appendix I.

The morphology of elements of the stylopodium and zeugopodium, excluding the autopodial elements, of the limbs was described considering form, size, and orientation. For a more comprehensive description, it was divided into A) forelimb: humerus, radius, and ulna; and B) hindlimb: femur, tibia, and fibula. The nomenclature for the anatomical description follows that of previous studies on different groups of mammals (Evans 1993; Argot 2001, 2002, 2003; Sargis 2001, 2002a, b; Horovitz and Sanchez-Villagra, 2003; Bezuidenhout and Evans 2005; Selthofer et al. 2006; Flores and Diaz 2009; Morgan and Verzi 2011). The anterior and posterior autopodium are not included because they are not complete or conserved in most analyzed specimens.

**Figure 1.**
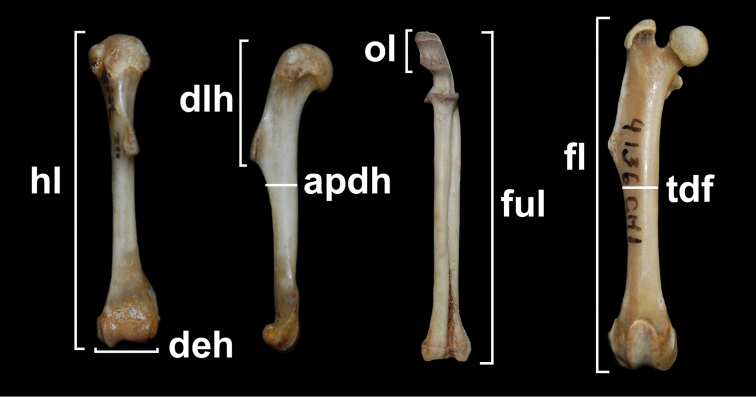
Measurements of the long bones. apdh, anteroposterior diameter of the humerus; deh, diameter of the epicondyles; dlh, deltoid length of the humerus; fl, functional femur length; hl, functional humerus length; ful, functional ulna length; ol, length of the olecranon process; tdf, transverse diameter of the femur.

Based on the previous researches by Biknevicius (1993), Elissamburu and Vizcaino (2004), Morgan and Verzi (2006), and Hopkins and Samuels (2009), eight measurements of the humerus, ulna, and femur (Fig. 1) corresponding to diameters and functional lengths (length among articular surfaces) of the bones and muscular insertion sites, were taken with digital calipers to the nearest 0.01 mm. Five indexes with functional significance, calculated from linear measurements were selected, based on a qualitative assessment and previous proposals (Bicknevicius et al. 1993; Fernandez et al. 2000; Elissamburu and Vizcaino 2004; Morgan and Verzi 2006). These indexes are: 1) Shoulder moment index (SMI): dlh/hl ? 100, where dlh is the deltoid length of the humerus and hl is the functional length of the humerus; this index is an indication of the mechanical advantage of the deltoid and major pectoral muscles acting across the shoulder joint; 2) Epicondyle index (EI): deh/hl ? 100, where deh is the epicondylar width of the humerus; this index is considered a good indicator of fossoriality; 3) Humeral robustness index (HWL): apdh/hl ? 100, where apdh is the anteroposterior diameter of the humerus; it is a good indicator of general resistance of the bone; 4) Index of fossorial ability (IFA): ol/(ful-ol) ? 100, ol is the length of the olecranon process and ful is the functional ulna length; this index gives a measure of the mechanical advantage of the triceps and dorsoepitrochlearis muscles in elbow extension, it is also a good indicator of fossoriality; 5) Femur robustness index (FRI): tdf/fl ? 100, where tdf is the transverse diameter of the femur; this index gives an idea of capacity for supporting body mass and to withstand the vertical forces associated with speed increase.

## Results

### Description

**Figure 2.**
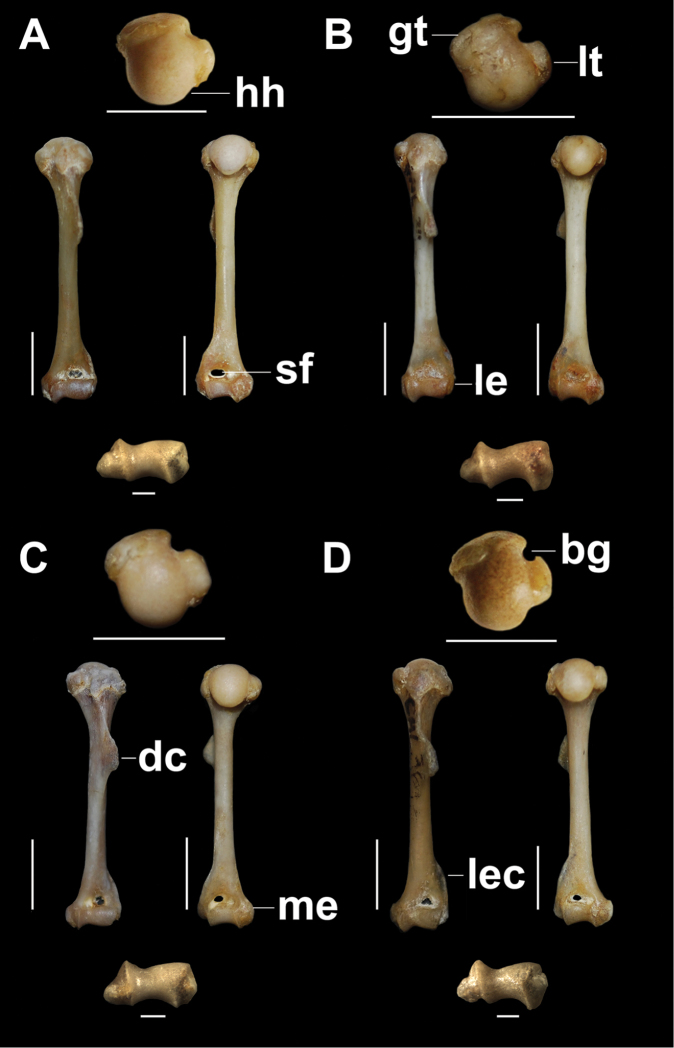
Left humerus, proximal, anterior, posterior, and distal views. **A** *Tympanoctomys aureus* **B** *Tympanoctomys barrerae* **C** *Tympanoctomys kirchnerorum* **D** *Tympanoctomys loschalchalerosorum*. bg, bicipital groove; dc, deltoid crest; gt, greater tuberosity; hh, humeral head; le, lateral epicondyle; lec, lateral epicondylar crest; lt, lesser tuberosity; me, medial epicondyle; sf, supratrochlear foramen. Scale bars 5 mm for all views except distal view where the scale bars are 1 mm.

***Humerus***. The diaphysis is robust with a cross-section angled in *T. loschalchalerosorum* and more cylindrical in the other species (Fig. 2). In all species of *Tympanoctomys*, the head is oval, elongated in the sagittal plane, and becomes narrower distally with a posterior extension forming a “peak” (Fig. 2). In *T. loschalchalerosorum* and *T. barrerae*, the greater and lesser tubercles are separated by a deep and narrow bicipital groove, which is shallower and broader in *T. aureus* and *T. kirchnerorum*. The greater tubercle is oval, has an irregular surface, posteriorly extended, and can be observed in caudal view, with a marked humeral lateral tuberosity. The lesser tubercle is oval and more extended toward the proximo-medial portion in *T. loschalchalerosorum*; furthermore, it is more developed in *T. kirchnerorum* than in the other species, and medially projected. In all analyzed species, the tubercles do not surpass the height of the humeral head (Fig. 2). In *T. loschalchalerosorum* and *T. barrerae*, a small foramen is located between the two tubercles.

The deltoid crest is located, in all species, in the proximal half portion of the diaphysis, it is well developed and greatly expanded laterally in *T. loschalchalerosorum* and *T. barrerae*, and ends as a pointed tip; whereas in *T. aureus* the distal tip is rounded and slightly extends laterally, it is more cranially oriented, similar to *T. kirchnerorum*. In the distal epiphysis, the capitulum is flattened, expanded, and separated from the trochlea by a well-marked groove. The trochlea is broader than the capitulum, is pulley-shaped, and its orientation with respect to the longitudinal axis of the humerus is straight in *T. barrerae* and oblique in the other species. The lateral epicondylar crest is well developed in *T. loschalchalerosorum* (Fig. 2C) in comparison to the other species, whereas the medial epicondyle is equal in size in all species. The supratrochlear foramen is observed in all specimens of all species analyzed (the greatest development was observed in *T. aureus*), except in specimens of *T. barrerae* in which it can be present or absent; when the foramen was completely ossified it was considered as absent. In *T. loschalchalerosorum* and *T. kirchnerorum*, the radial and olecranon fossa are shallow, whereas in *T. aureus* the radial fossa, and in *T. barrerae* the olecranon fossa, are the deepest. In all species analyzed, the entepicondylar foramen is absent and a notch on the articular surface with the radius is observed.

**Figure 3.**
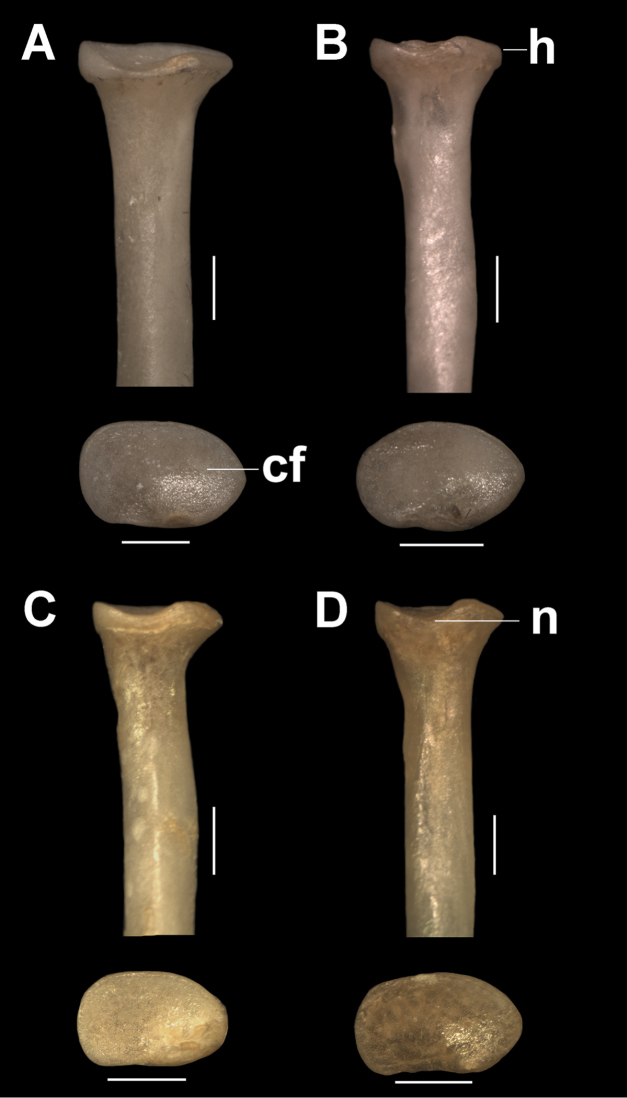
Cranial and proximal views of the proximal portion of the right radio. **A** *Tympanoctomys aureus* **B** *Tympanoctomys barrerae* **C** *Tympanoctomys kirchnerorum* **D** *Tympanoctomys loschalchalerosorum*. cf, central fossa; h, head; n, notch. Scale bars 1mm.

**Figure 4.**
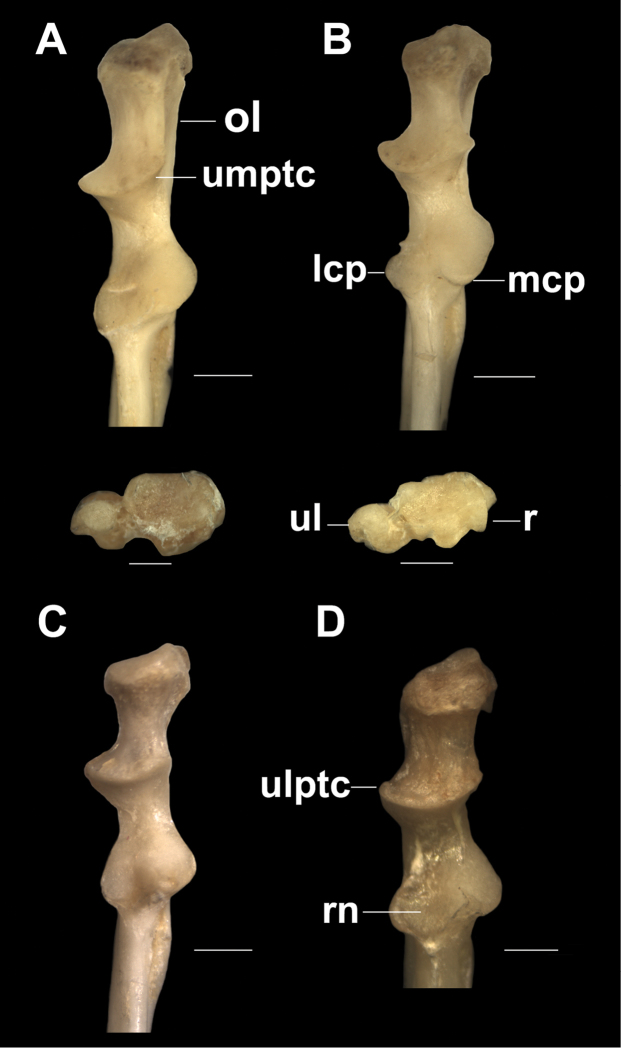
Right ulna, proximal portion in cranial and distal views of radio and ulna. **A** *Tympanoctomys aureus* **B** *Tympanoctomys barrerae* **C** *Tympanoctomys kirchnerorum* **D** *Tympanoctomys loschalchalerosorum*. lcp, lateral coronoid process; mcp, medial coronoid process; ol, olecranon; r, radio distal surface; rn, radial notch; ul, ulnar distal surface; ulptc, ulnar lateral proximal trochlear crest; umptc, ulnar medial proximal trochlear crest. Scale bars 1 mm.

***Radius and ulna.*** In *T. loschalchalerosorum*, *T. barrerae*, and *T. kirchnerorum* the diaphysis of the radius is somewhat cylindrical, with a flat side on the contact surface with the ulna, whereas in *T. aureus* it is more compressed (Figs 3, 4). In the proximal epiphysis, the central fossa of the radius is oval and concave in all species. The anterior edge of the proximal epiphysis (opposed surface to the articulation with the ulna) has a notch (Fig. 3) clearly evident in *T. loschalchalerosorum* and *T. kirchnerorum*, less evident in *T. aureus*, and almost imperceptible in *T. barrerae*. The articular fovea is well-marked and oval in all species, with a less concave surface in *T. aureus* and *T. barrerae*. The neck is well marked, more evident in *T. kirchnerorum*. The radial tuberosity is well developed in all species except in *T. barrerae*. The proximal portion of the diaphysis is slightly curved cranially, and the posterior proximal border is straight.

In the ulna (Fig. 4), the olecranon is short, more robust in *T. loschalchalerosorum* (Fig. 4D) and thinner in *T. kirchnerorum* (Fig. 4C) than in the other species. The anconeal process is poorly developed and defined by two small crests: the ulnar lateral proximal trochlear crest (ulptc) and the ulnar medial proximal trochlear crest (umptc). The umptc varies among specimens of *T. aureus*; in the holotype (CML 6137) is similar to the other three species where the angle is not greater than 30°, while in the paratype (CML 4137) and other examined specimens from the CMI, this crest is markedly oblique at an angle of 45° with respect the body axis of the ulna. The trochlear notch is relatively open. The lateral coronoid process is well developed in *T. barrerae* and the holotype of *T. aureus*, less developed in *T. loschalchalerosorum* and *T. kirchnerorum*, and tiny in the paratypes and other specimens examined of *T. aureus*. The medial coronoid process protrudes anteriorly and is less developed in some specimens of *T. aureus*. The radial notch is wide and concave in all species; in *T. loschalchalerosorum* and *T. barrerae* is rounded and oblique, in *T. aureus* it is less oblique and in *T. kirchnerorum* it is almost horizontal (Fig. 4). The lateral fossa is deep in all species.

The distal epiphysis of the radius and ulna are here described for only two species (*T. aureus* and *T. barrerae*), because in other species these structures were broken and missing in the specimens examined. The medial styloid process of the radius is poorly developed in both species; the carpal surface is more concave and triangular in *T. barrerae*, whereas it is crescent-shaped in *T. aureus*. The medial styloid process of the ulna is well developed in both species, being proportionally longer in *T. barrerae*, and rounder in *T. aureus* (Fig. 4)

**Figure 5.**
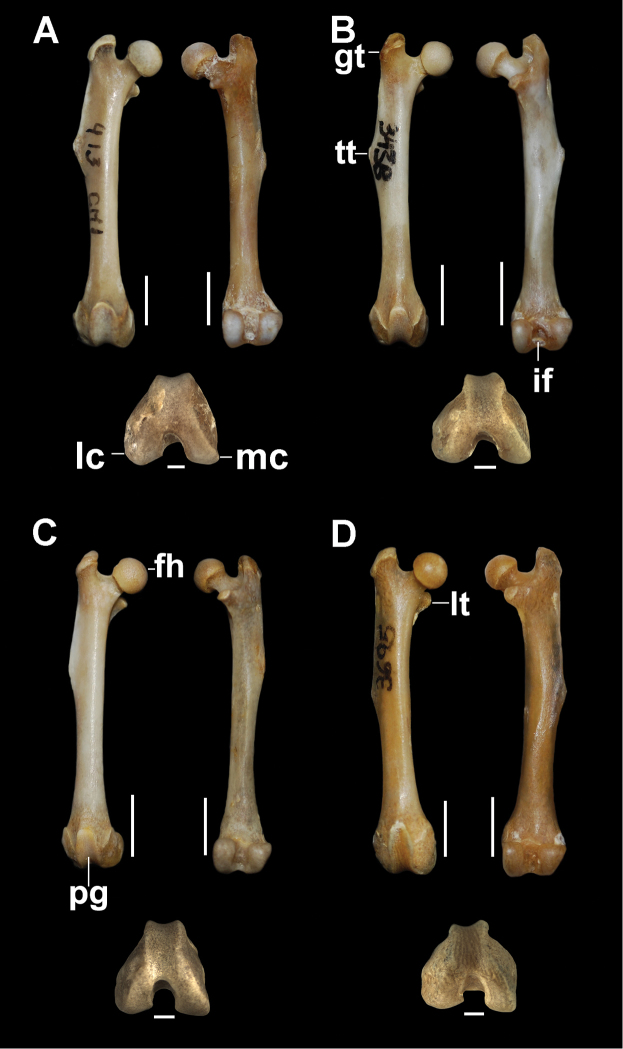
Right femur, anterior, posterior, and distal views. **A** *Tympanoctomys aureus* **B** *Tympanoctomys barrerae* **C** *Tympanoctomys kirchnerorum* **D** *Tympanoctomys loschalchalerosorum*. fh, femoral head; gt, greater trochanter; if, intercondylar fossa; lc, lateral condyle; lt, lesser trochanter; mc, medial condyle; pg, patellar groove; tt, third trochanter. Scale bars 5 mm for all views except distal view where the scale bars are 1 mm.

***Femur.*** The femur is robust with a straight and cylindrical diaphysis. In the proximal epiphysis, the femoral head is spherical and dorso-medially oriented with a short neck (Fig. 5). The greater and lesser trochanters are well developed; the greater trochanter slightly extends dorsally above the head. The lesser trochanter is postero-medially oriented. The third trochanter is poorly developed in *T. loschalchalerosorum*, *T. barrerae*, and *T. kirchnerorum*, and more developed in *T. aureus*. In *T. loschalchalerosorum* the third trochanter lies in the middle of the diaphysis, whereas in *T. barrerae, T. aureus*, and *T. kirchnerorum* it is more proximally located. The trochanteric fossa is well developed in all species, but is deeper in *T. aureus*.

In the distal epiphysis, the lateral condyle is slightly wider than the medial condyle, which is more distally projected. The intercondylar fossa is narrow and deep, being shallower in *T. aureus* and *T. barrerae* than the other two species. The patellar groove is narrow and bordered by two parallel ridges.

**Figure 6.**
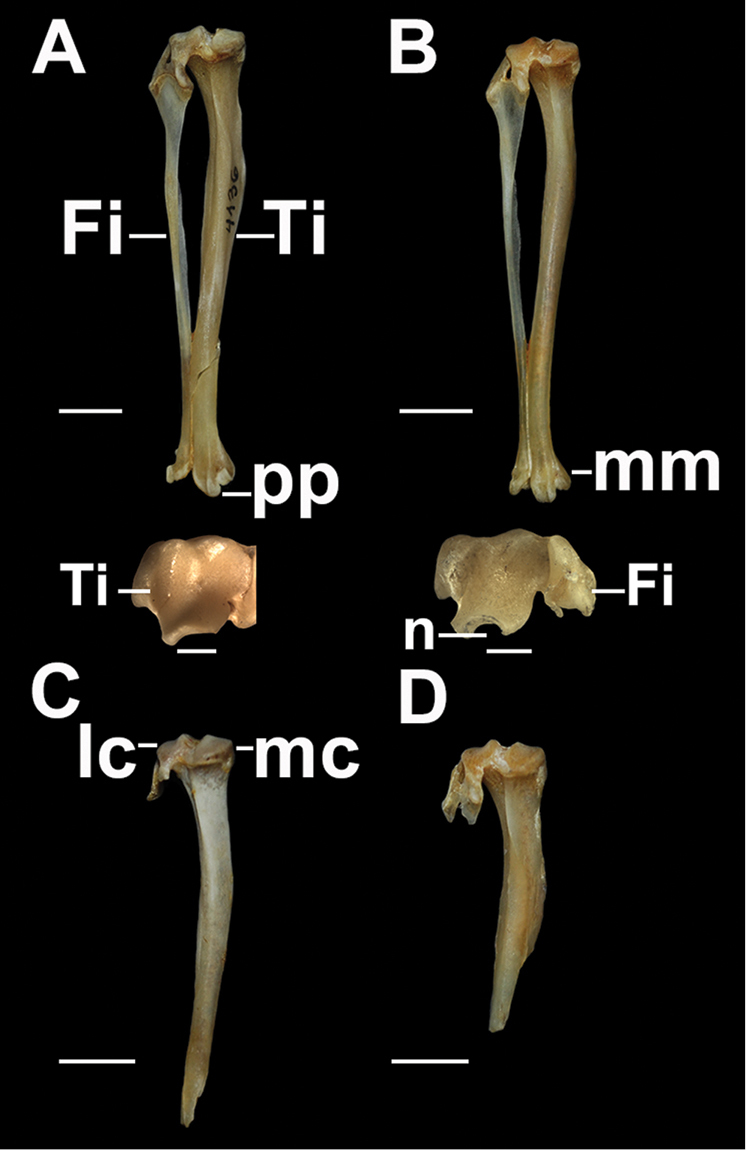
Caudal and distal views of the right tibia and fibula. **A** *Tympanoctomys aureus* **B** *Tympanoctomys barrerae* **C** *Tympanoctomys kirchnerorum* **D** *Tympanoctomys loschalchalerosorum*. Fi, fibula; lc, lateral condyle; mc, medial condyle; mm, medial malleolus; n, notch; pp, posterior process; Ti, tibia. Scale bars 5 mm for all views except distal view where the scale bars are 1 mm.

**Figure 7.**
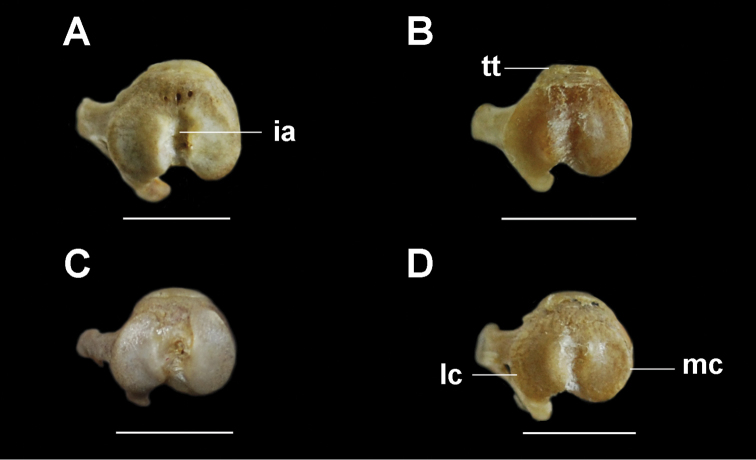
Proximal view of the tibia. **A** *Tympanoctomys aureus* **B** *Tympanoctomys barrerae* **C** *Tympanoctomys kirchnerorum* **D** *Tympanoctomys loschalchalerosorum*. a, intercondylar area; lc, lateral condyle; mc, medial condyle; tt, tibial tuberosity. Scale bars 5mm.

***Tibia and fibula.*** In the analyzed specimens of *T. loschalchalerosorum* and *T. kirchnerorum*, the tibias were broken and distal epiphyses were missing, so that many of the characters could not be described (Figs 6, 7). In *T. aureus* and *T. barrerae*, the tibia is approximately 25% longer than the femur. The lateral and medial condyles are oval; in *T. loschalchalerosorum*, they are sub-equal in size (Fig. 7D), whereas in the other species the lateral condyle is slightly larger than the medial condyle. In *T. loschalchalerosorum*, the two condyles are slightly concave, and tend to be flattened; in *T. barrerae* and *T. kirchnerorum* the lateral condyle is slightly more concave than the medial, and in *T. aureus* the medial condyle is flatter than the lateral (Fig. 7). The lateral condyle is somewhat higher than the medial and has a caudal projection. In *T. loschalchalerosorum* and *T. barrerae*, the intercondylar area is narrow and concave with an evident groove; this intercondylar area is caudally wider in *T. aureus*, and wider and deeper in *T. kirchnerorum*. The popliteal notch is narrow with a well-marked fossa in *T. loschalchalerosorum* and *T. barrerae*, broad and shallower in *T. aureus*, and narrow and deeper in *T. kirchnerorum*. The tibial tuberosity is developed and anteriorly projected below the condyles. In *T. loschalchalerosorum*, the tibial crest is more strongly developed and antero-medially extended, than in the other species, and in *T. barrerae* the crest is located slightly closer to the proximal tip. A caudal crest, observed between the condyles and the popliteal notch, is well developed in *T. loschalchalerosorum*, less developed in *T. barrerae* and *T. kirchnerorum*, and absent in *T. aureus*.

In *T. aureus*, the two foveae on the distal epiphysis are oval, and the medial fovea is narrower and more concave than the lateral one (Fig. 6A). In *T. barrerae* (Fig. 6B), these foveae are more rounded and the medial is also deeper than the lateral, but not as much as in *T. aureus*. The ridge that divides the two foveae is well developed in *T. aureus* and less developed in *T. barrerae*. The medial malleolus is sub-quadrangular, short, and transversally wide in *T. barrerae* and slightly longer and sub-triangular in *T. aureus*. Distal edge of medial malleolus is more pointed in *T. barrerae* than *T. aureus*. In both species, the posterior process and the groove for tendon of *flexor digitorum tibialis* are evident; the posterior edge of the process is concave, but in *T. barrerae* a marked notch is observed (Fig. 6A, B).

The fibula (Fig. 6) shows the distal portion cylindrical and the proximal portion compressed. The head is fan-shaped. In the proximal union with the tibia, a well-developed foramen is observed. The lateral fibular malleolus is more rounded in *T. barrerae* than in the other species, whereas it is slightly elongated with an irregular surface in *T. aureus*.

***Indexes.*** For each species, the results are indicated in Table 1. In the humerus, the averages for the genus are: SMI=42.65, EI=21.6, and HWL=8.76. *Tympanoctomys aureus* shows the highest values of SMI, HWL, and EI. *Tympanoctomys kirchnerorum* has the lowest SMI while *T. loschalchalerosorum* has the lowest HWL. For the ulna, IFA average is 11.56, values are the highest in *T. aureus*, followed by *T. barrerae*, *T. kirchnerorum* and *T. loschalchalerosorum* with the lowest. Finally, in the femur, for FRI the average is 10.03, with the lowest value in *T. kirchnerorum* and the highest in *T. loschalchalerosorum*. Additionally, these indexes were also calculated for *C. opimus* for comparisons with the semifossorial *Tympanoctomys*, and although it has the highest values in most of the indexes, it is worth noting the value of the epicondylar development (EI) of the humerus as well as the fossorial ability (IFA). This analysis is preliminary and aims to help in the description and comparison among species.

**Table 1.** Mean, standard deviation, and number of specimens in brackets are indicated for each index and species.

	*T. aureus*	*T. barrerae*	*T. kirchnerorum*	*T. loschalchalerosorum*	*Ct. opimus*
SMI	44.43 1.76 (21)	42.96 2.05 (30)	41.44 1.52 (2)	41.77 - (1)	51.45 2.55 (4)
HWL	9.46 0.73 (21)	9.04 0.57 (30)	8.71 0.12 (2)	7.86 - (1)	11.1 0.62 (4)
EI	22.54 0.9 (21)	21 1.3 (30)	21.55 0.58 (2)	21.28 - (1)	30 0.8 (4)
IFA	15.71 3.2 (2)	13.02 1 (4)	9.02 0.02 (2)	8.51 - (1)	20 0.5 (3)
FRI	9.66 0.55 (7)	9.65 0.53 (28)	9.18 0.66 (2)	11.41 - (1)	10.27 0.8 (4)

## Discussion

Generally, most studies have been limited to one species of *Tympanoctomys* (*T. barrerae)* therefore there is almost no information about morphological change within species. This study provides a detailed description of the postcranial elements of the limbs of all species of the genus *Tympanoctomys* including the holotype of *T. loschalchalerosorum*, one of the only two known specimens of this species in the world, and the recently described *T. kirchnerorum*. As taxonomists know, using descriptions based on a single specimen is not the best protocol; nevertheless, it is useful in phylogenetic reconstructions. Because this unique material is not available in systematic collections and the animals are very rare in natural environments, the descriptions presented here represent the first qualitative approach for the species of the genus *Tympanoctomys* and for the genus as such. The information here would be useful for future comparisons with the rest of the octodontids.

The morphological differences among the species of the genus are still under revision. Therefore, here is the importance of including new evidence, such as postcranial characters in phylogenetic analysis. Mares et al. (2000), based on external and cranial morphology, suggested a greater affinity between *T. loschalchalerosorum* and *T. aureus*. However, when a fourth species (*T. kirchnerorum*) was included in the analysis, that was not previously considered, it might be expected that the relationship among species would be affected. These results show that *T. loschalchalerosorum* shares more attributes (e.g. bicipital groove of the humerus, intercondylar area, and popliteal notch of the tibia) with *T. barrerae* than with the other species. Furthermore, considering the remainder of the postcranial elements, *T. aureus* shows more affinity with *T. kirchnerorum* than with the other species (data unpublished and under analysis data from Perez 2013).

On the other hand, the postcranial elements of the forelimbs and hind limbs of the four species of the genus show an anatomical plan related to the mode of terrestrial life, consistent with what was observed in other rodents and marsupials, for example the posterior extension of the humeral head forming a “peak”, the tubercles not surpassing the head, the separation between the trochlea and the capitulum, the flat or just concave articular surface of the radial notch in the ulna, the diaphysis of the radio curved, the extension of the greater trochanter above the femoral head, the posterior or posteromedial position of the third trochanter, and the asymmetry between the lateral and medial condyles, with the lateral wider (Hatt 1932; Sargis 2002a, b; Argot 2003; Candela and Picasso 2008; Flores and Diaz 2009; Olivares 2009; Carrizo and Diaz 2011). However, because the genus *Tympanoctomys* is semi-fossorial and scratch digging, characteristics in their long bones were expected which reflected their digging habit (Elissamburu and Vizcaino 2004; Morgan and Verzi 2006; Samuels and Van Valkenburgh 2008, 2009; Salton and Sargis 2008; Hopkins and Davis 2009). Previous studies (e.g. Morgan and Verzi 2006; Lessa et al. 2008) indicate that *Tympanoctomys* as well as *Octodon* and *Aconaemys* are capable of building complex burrows, consisting in oblique tunnels that connect the surface with their nests, including several bifurcations and openings, but in *T. aureus* the tunnels are nearly parallel to the ground surface (M.M. Diaz and R.M. Barquez personal observations). Contrary to what were expected in semi-fossorial species, these rodents have slender humerus, radius and ulna; narrow epicondyles of the humerus and short olecranon of the ulna with poorly developed processes.

Almost all indexes analyzed in *Tympanoctomys* have values below those calculated in *Ctenomys*, except for the robustness of the femur where *T. loschalchalerosorum* shows a higher value. Moreover, the results for SMI, HWL, EI and IFA in *T. aureus* has higher values among *Tympanoctomys*. These scores and a lowest value for the robustness of the femur can be related with allometric changes in *T. aureus*, the largest species of the genus. Elissamburu and Vizcaino, (2004) and Elissamburu and De Santis (2011) analyzed the morphometric variation in other caviomorph rodents to evaluate the fossorial forms in a functional context. Comparing our results, the loadings of the indexes in *Tympanoctomys* are more related to cursorial forms or occasional diggers, like *Dolichotis* or *Microcavia*. Although our analysis is preliminary and serves to quantify what is qualitatively described; in the future deeper studies will be performed including all octodontid taxa.

Lessa et al. (2008), who analyzed and compared certain muscle-skeletal characteristics of Octodontidae, concluded that neither *Octomys* nor *Tympanoctomys* (including only *T. barrerae*) shows great skeletal adaptations related with digging capacity. The octodontid and ctenomyid rodents, two closely related families, are included within the five extant families of rodents (Geomyidae, Ctenomyidae, Octodontidae, Bathyergidae, and Muridae, including Spalacinae and Rhizomyinae) in which the fossorial and subterranean habits have evolved independently, as a further specialization in close association with the emergence of open environments during mid to late Cenozoic (Lessa et al. 2008). This is especially interesting and encourages the development of studies to learn more the about behavioral and structural adaptations in these families of rodents. Some authors (Elissamburu and Vizcaino 2004; Morgan and Verzi 2006; Elissamburu and De Santis 2011; Morgan and Alvarez 2013) have studied the adaptations of the forelimbs and hindlimbs, especially the digging capacity of *Ctenomys*, and concluded that the greater development of the medial epicondyle could be an early specialization and one of the main characters by which to recognize the digging fossorial forms. In *Tympanoctomys*, this feature is not observed; it can be related to the fact that this genus occurs in sandy soils then strong modifications of the limbs are not necessary.

Although these results are preliminary, data obtained in this study are consistent with observations made by other authors for other members of the family Octodontidae, and provide information for species that are poorly known or recently described. The family Octodontidae is highly specialized and adapted to living in desert habitats with a wide range of habits in just a few genera, so it is expected that the limbs have modified structures for that purpose. According to our descriptions, there are not too many intrageneric morphological variations regarding to the configuration of the limbs, probably due to phylogenetic proximity, and not related to specific variations in response to different use of substrates or habits. With regard to ecological aspects, just one species (*T. barrerae*) is well known about their burrow system, which is considered as an intermediate level of complexity compared with *Ctenomys* (see Lessa et al. 2008), and something similar was observed by two of the authors (RMB and MMD) in *T. aureus*; nothing is known for the other two species.

Some authors (Upham and Patterson 2012, 2015; Suarez-Villota et al. 2016), through molecular studies, suggested to maintain the four species under the same genus. But Suarez-Villota et al. (2016), in their analysis, do not recognize *T. loschalchalerosorum* as a valid taxon but included within the diversity of *T. barrerae*. As mentioned above, *T. loschalchalerosorum* and *T. barrerae* share several characteristics, but also similarities are observed between *T. aureus* and *T. kirchnerorum* (e.g, shape and orientation of the deltoid crest in the humerus) and *T. aureus* and *T. barrerae* (e.g. depth of intercondylar fossa of the femur). We are currently studying the rest of the postcranial morphology in *Tympanoctomys* like in the other members of the family Octodontidae, in order to understand their evolution in relation to the huge eco-morphological diversity and their origin and time of diversification. Following these proposal, it would be interesting to include information of postcranial morphology as new evidence in comprehensive phylogenetic analysis with all the octodontid members.

## Appendix

List of specimens analyzed detailing the number of individuals by species in brackets, collection localities, type specimens, and collection numbers are indicated. See materials and methods by collections acronyms.

*Ctenomys opimus* (4): Jujuy Province, Susques Department: Borde de Ecalon, 31 km al SO de Susques, por ruta 52 y 1 km SE de ruta, 1 (CML 9353). Salta Province, Los Andes Department: Vega Cortadera 1 km W de ruta 53, 3910 m, 2 (CML 7243, 7244); 36 km N San Antonio de Los Cobres, 11,600 ft., 1 (CML 8438).

*Tympanoctomys aureus* (22): Catamarca Province, Poman Department: Salar de Pipanaco, 10 km de Pio Brizuela (Est. Rio Blanco), km 96 sobre R46, 35 km S de Andalgala, 3 (CMI 6565, 6818, 6888); en los bordes del Salar Pipanaco, 3 (CMI 6562, 6563, 6564); 5 km del puesto de Pio Brizuela, entrada km 96 sobre R46, 2 (CMI 7188, 7189); a 13 km de la entrada Establecimiento Rio Blanco, 4 (CMI 6558, 6559, 6560, 6561); Establecimiento Rio Blanco, 28 km S, 9.3 km W Andalgala, 4 (CML 6137, holotype; 4136, 4137, paratypes; 10110); Pipanaco, Salar Pipanaco, 6 (CMI 6846, 6848, 6849, 6850, 6851, 6856).

*Tympanoctomys barrerae* (35): La Pampa Province, Limay Mahuida Department: Gran salitral, 6 (CMI 6877, 6878, 6879, 6880, 6882, 6883). Mendoza Province, La Paz Department: 27 km N Desaguadero, 556 m app, 2(CMI 4485, CML 3438), Desaguadero, El Tapon 37 km, 1 (CMI 3314); Lavalle Department: 34 km al N de Desaguadero camino a Arroyito, 1 (CML 10111); Malargue Department, 6(CMI 7263, 7264, 7266, 7267, 7268, 7269), a 8.5 km camino a Llancanelo, 1(CMI 7098), Camino Llancanelo 7(CMI 7270, 7271, 7272, 7273, 7274, 7275, 7276), Laguna Llancanelo, 2(CMI 7248, 7250); San Rafael Department: 10 km S El Nihuil 2 (CMI 3845, 3846), El Nihuil, 2 (CMI 6290, 6291). San Juan Province, Valle Fertil Department: Parque Provincial Ischigualasto, 4 (CMI 6842, 6843, 6853, 6889). San Luis Province, 1 (CMI 3821).

*Tympanoctomys loschalchalerosorum* (1): La Rioja Province, Chamical Department: 26 km SW Quimilo, 1 (CML 3695, holotype).

*Tympanoctomys kirchnerorum* (2): Chubut Province, Sarmiento Department: Ea. La Porfia, 2 (CNP 2503, 2505).
